# Electromagnetic Interference in the Modern Era: Concerns, Trends, and Nanomaterial-Based Solutions

**DOI:** 10.3390/nano15201558

**Published:** 2025-10-13

**Authors:** Jovana Prekodravac Filipovic, Mila Milenkovic, Dejan Kepic, Sladjana Dorontic, Muhammad Yasir, Blaz Nardin, Svetlana Jovanovic

**Affiliations:** 1 Vinca Institute of Nuclear Sciences-National Institute of the Republic of Serbia, University of Belgrade, Mike Petrovica Alasa, 12-14, 11351 Belgrade, Serbia; mila.milenkovic@vin.bg.ac.rs (M.M.); d.kepic@vin.bg.ac.rs (D.K.); sladjana.dorontic@vin.bg.ac.rs (S.D.); 2 Division of Microrobotics and Control Engineering, Department of Computing Science, Carl von Ossietzky Universität Oldenburg, 26129 Oldenburg, Germany; muhammad.yasir@uni-oldenburg.de; 3 Faculty of Polymer Technology, Ozare 19, 2380 Slovenj Gradec, Slovenia; blaz.nardin@ftpo.eu

**Keywords:** electromagnetic interference (EMI), shielding materials, carbon-based nanomaterials, graphene, graphene oxide, sustainable nanocomposites, wireless technologies

## Abstract

Electromagnetic interference (EMI) represents a growing challenge in the modern era, as electronic systems and wireless technologies become increasingly integrated into daily life. This review provides a comprehensive overview of EMI, beginning with its historical evolution over centuries, from early power transmission systems and industrial machinery to today’s complex environment shaped by IoT, 5G, smart devices, and autonomous technologies. The diverse sources of EMI and their wide-ranging effects are examined, including disruptions in electrical and medical devices, ecological impacts on wildlife, and potential risks to human health. Beyond its technical and societal implications, the economic dimension of EMI is explored, highlighting the rapid expansion of the global shielding materials market and its forecasted growth driven by telecommunications, automotive, aerospace, and healthcare sectors. Preventative strategies against EMI are discussed, with particular emphasis on the role of advanced materials. Carbon-based nanomaterials—such as graphene, carbon nanotubes, and carbon foams—are presented as promising solutions owing to their exceptional conductivity, mechanical strength, tunable structure, and environmental sustainability. By uniting perspectives on EMI’s origins, consequences, market dynamics, and mitigation strategies, this work underscores the urgent need for scalable, high-performance, and eco-friendly shielding approaches. Special attention is given to recent advances in carbon-based nanomaterials, which are poised to play a transformative role in ensuring the safety, reliability, and sustainability of future electronic technologies.

## 1. Introduction

Electromagnetic interference (EMI) refers to the disruption of the normal operation of electronic devices caused by electromagnetic radiation originating from both natural and man-made sources. EMI shielding is quantified by shielding effectiveness (SE), defined as the logarithmic ratio of incident to transmitted electromagnetic power, expressed in decibels (dB). It captures the combined effects of reflection, absorption, and multiple internal reflections [1]. For most commercial electronics, a typical SE range is 40–60 dB, which corresponds to a 99.99–99.999% attenuation of interfering radiation [2]. Higher values (80–100 dB or more) are required in more demanding industrial, medical, or military applications. As modern society grows increasingly dependent on electronic systems and wireless communications, the sources and complexity of EMI have evolved significantly. Initially, EMI was primarily associated with power lines, radio transmissions, and industrial machinery. However, in recent decades, it has expanded to include a wide array of everyday technologies [1].

The evolution of EMI is tightly coupled through the centuries with the exponential growth of electronic devices and high-frequency applications. With the rise of the Internet of Things (IoT), smart homes, autonomous vehicles, and 5G technologies, electromagnetic pollution has become more prevalent and intricate. These developments have led to an increase in both intentional emissions—such as from communication devices—and unintentional emissions from electronic circuits, switching power supplies, and digital processors [3].

The consequences of EMI are broad and significant. In the healthcare sector, EMI can compromise the functionality of active medical implants such as pacemakers, defibrillators, and insulin pumps, posing direct threats to patient safety. In the function of consumer electronics, EMI contributes to malfunctions, reduced signal integrity, and shortened device lifespans. Additionally, the potential effects of long-term electromagnetic exposure on human health—including stress, sleep disturbances, and possible links to certain illnesses—have raised public concern. EMI also impacts the environment, particularly through its interference with wildlife navigation systems and its contribution to ambient electromagnetic pollution in urban areas [4,5,6,7].

From an economic perspective, the EMI shielding market has grown rapidly in response to these concerns. It is forecasted to reach multi-billion-euro valuations in the coming years, driven by the need to protect sensitive electronic components across sectors such as telecommunications, automotive, aerospace, healthcare, and defense. As awareness of EMI-related risks grows, so too does the demand for effective, lightweight, and sustainable shielding materials [8,9].

At industrial scale, materials currently in use include metals (e.g., copper, aluminum, nickel) owing to their high conductivity; metal-coated polymers or foams offering lighter weight and flexibility; thermoset and thermoplastic matrices such as epoxy, polyurethane, polypropylene, PEEK, PPS, ABS hosting conductive or magnetic fillers; and carbon-based particulates (graphite, carbon black, carbon nanotubes) and hybrid fillers. For example, a study on metal-coated polymeric materials employing PPS, PEEK, and PPA matrices with coatings of Zn or Al-Bronze achieved SE values rising from ~7 dB (uncoated) to ~40–60 dB with coating in frequencies across low to high bands [10]. Additionally, polymer nanocomposites with carbon-based fillers have been reported with SE up to ~91.9 dB in laboratory settings [11]. One of the most promising classes of materials for EMI shielding is carbon-based nanomaterials [12,13], including graphene, carbon nanotubes (CNTs), and carbon foams, among others. These materials exhibit exceptional electrical conductivity, mechanical strength, thermal stability, and chemical resistance, making them highly effective in absorbing and reflecting electromagnetic waves. In particular, advanced carbon-based foams offer tunable porosity and large surface areas, enhancing their capacity for EMI attenuation while maintaining low density and structural flexibility [12,14,15,16]. Comparisons between micron-sized fillers (e.g., graphite particles, metallic spheres) and metallic particles such as copper reveal important trade-offs. While copper offers very high intrinsic electrical conductivity (thus good reflection), micron graphite or carbon particulates may outperform in terms of specific shielding effectiveness (i.e., per unit weight or thickness), better corrosion resistance, lower density, enhanced absorption losses, and improved impedance matching at high frequencies. These factors often lead to superior performance in applications where weight, flexibility, or form factor are important. These findings are supported by reviews and studies that show carbon filler composites can achieve moderate to high SE (~40–80 dB) under optimized conditions [11].

The advantages of nanocomposites over micro-scale or conventional fillers stem from several mechanisms:

- Lower percolation thresholds due to the high aspect ratio or sheet-like geometry of nanofillers (e.g., CNTs, graphene), enabling conductive networks at lower volume fractions.
- Increased surface area, which enhances interfacial polarization, dielectric losses, and scattering of incident EM waves (thus attenuation via absorption) [17].
- Ability to engineer multi-functional architectures (foams, laminates, hybrids) that combine reflective and absorptive shielding, while maintaining lower weight and thinner profiles suitable for practical industrial usage [18].

Within the European Union, various initiatives and regulatory frameworks have been established to encourage the development and adoption of environmentally friendly and high-performance shielding solutions [19]. Research consortia and innovation hubs across Europe are actively engaged in creating scalable, cost-effective, and sustainable carbon-based materials tailored for EMI protection in next-generation electronics [20].

Preventative measures against EMI now encompass not only regulatory compliance and device design optimization but also the strategic integration of shielding materials during product development. As we move further into the era of ubiquitous connectivity and electronic integration, the role of advanced carbon-based EMI shielding solutions will be critical in ensuring the safety, reliability, and environmental sustainability of future technology.

This review addresses critical literature gaps by examining both natural and artificial sources of electromagnetic interference (EMI) and tracing its progression over centuries in parallel with technological development. Particular emphasis is placed on the long-term implications of electromagnetic exposure, highlighting its impact on the healthcare sector, ecological systems, and the reliable operation of electronic and medical devices. Beyond technical and societal effects, the review also analyzes the economic dimension, outlining the rapid growth and future trajectory of the EMI shielding market.

Given the exponential advancement of materials science and technology, a wide range of shielding materials has been developed; however, many challenges remain in achieving scalable, lightweight, and environmentally sustainable solutions. By carefully evaluating the current body of research, this work provides an in-depth assessment of state-of-the-art carbon-based nanomaterials—such as graphene, carbon nanotubes, and carbon foams—underscoring their potential as high-performance, next-generation shielding materials. In doing so, this review not only consolidates existing knowledge but also offers valuable insights into the most promising research directions for sustainable EMI mitigation.

## 2. EMI Source and Evolution

The concept of electromagnetic interference (EMI) shielding effectiveness (SE) denotes blocking the electromagnetic wave propagation through the barrier. EMI shielding materials are designed to block or non-ionizing EMWs (between 0 and 300 GHz), while most attention was focused on the extremely low, low, micro, and radio waves. It is crucial to differentiate EMI shielding from magnetic shielding, as the latter pertains to protection against a magnetic field, typically at low frequencies (such as 60 Hz) [21].

Electronic equipment creates RWs (3 kHz to 300 GHz) and MW (300 MHz to 300 GHz), particularly those functioning in the RWs and MW frequency bands, such as cell phones. The concern is that such waves disrupt electronic networks and other functions due to the interaction between the electrons in the metal conductor and the electric field within the radiation, leading to electronic malfunctions. The demand for further EMI shields and integrated circuit technology is escalating due to the proliferation of gadgets utilizing radio and MW frequencies. Consequently, extensive research into the fabrication of EMI shields has rapidly increased in recent years [22].

### 2.1. Sources of EMW

EMW pollution, also referred to as EMI, originates from both cosmic events and human actions. Before human interference, the Earth’s EM environment was influenced by natural events that generated constant and predictable EMW. These sources set a baseline for the planet’s EM spectrum, crucial for sustainable development and natural processes. Natural sources such as lightning and atmospheric discharges, solar radiation, cosmic rays, stellar events, and interactions with the Earth’s geomagnetic field have been significant but relatively constant throughout history, and thus have not played a major role in the environmental pollution accumulated in the modern era. The exponential proliferation of anthropogenic electromagnetic wave sources during the past century has significantly transformed the electromagnetic environment [23,24,25,26].

Anthropogenic electromagnetic interference arises from diverse systems and gadgets essential to contemporary existence, encompassing power grids, wireless communication networks, consumer electronics, and industrial machines [27,28,29]. These sources impact electronic systems and also elicit fears regarding their environmental and health ramifications [30]. The increase in human-induced electromagnetic interference is directly linked to the escalating dependence on technology in nearly all facets of society. The electromagnetic spectrum is fundamental to contemporary society, facilitating seamless wireless connectivity and enhancing the operational efficiency of industrial processes. This reliance presents concerns, including the potential for interference with essential systems, such as aircraft communication, medical devices, and navigation technologies [31]. Solar energy comprises electromagnetic radiation spanning the entire spectrum of wavelengths. Approximately 49% of this radiation lies in the low-frequency (long-wavelength) range, 44% falls within the visible spectrum perceptible to the human eye, and the remaining 7% is in the high-frequency (short-wavelength) ultraviolet range. The ultraviolet portion, being ionizing in nature, poses risks to human skin, eyes, brain, and other tissues. Additionally, solar activity such as flares and geomagnetic storms emits intense electromagnetic radiation that can significantly affect the Earth’s ionosphere and magnetosphere [23]. Lightning strikes are a major natural source of electromagnetic wave phenomena, generating broadband electromagnetic pulses. These events release significant electromagnetic energy primarily within the radio frequency range, spanning from below 1 Hz up to approximately 300 MHz. For lightning occurring at distances greater than 50 km, the peak emission typically falls between 5 and 10 kHz. The Earth’s magnetic field is a continuous source of low-frequency electromagnetic waves, especially during geomagnetic storms triggered by solar activity. Auroras, formed by solar particles interacting with the magnetic field, also emit across multiple frequencies. Cosmic radiation, originating from space, produces secondary EM emissions upon entering the atmosphere, potentially affecting communications and sensitive electronics. Additionally, volcanic eruptions and seismic events release detectable electromagnetic pulses, which are being studied for their potential in earthquake prediction and monitoring [26].

Wireless communication systems—such as cellular networks, Wi-Fi, and satellites—are major sources of electromagnetic wave pollution. The introduction of 5G, with its frequency and dense deployment of small cells, has intensified electromagnetic exposure, particularly in urban areas. Operating in the 24–100 GHz wave range, 5G networks increase EM field density and complexity, with signals reflecting, refracting, and diffracting off buildings and other surfaces, creating localized electromagnetic hotspots [28,32]. The IoT has introduced billions of interconnected devices, each emitting low-power radio frequency signals that cooperatively increase electromagnetic density. As a result, the IoT has become a significant source of electromagnetic emissions in both residential and professional/working environments [33,34]. Devices like smart meters, virtual assistants, and electronic wearables continuously emit EM waves, making them susceptible to EMI. EMI could disrupt communication, corrupt data, degrade system performance, and pose security risks. External sources—such as nearby electronic equipment or RF signals—can impair data transmission between IoT devices and gateways, reducing network reliability and responsiveness, and compromising the execution of critical functions. EMWs are essential to modern life, enabling many daily technologies. However, the widespread use of power lines, electrical wiring, and household appliances has raised concerns about their contribution to electromagnetic pollution. Most of these systems operate using alternating current (AC) at frequencies of 50 or 60 Hz, placing their emissions within the Extremely Low Frequency (ELF) range, defined as below 300 Hz [35]. Household appliances are common sources of electromagnetic emissions, with a wide range of frequencies depending on the type of device and its operating principle. For example, devices like microwave ovens operate at 2.45 GHz, Wi-Fi routers, smart TVs, and IoT-enabled appliances emit at 2.4 GHz and 5 GHz bands, while cordless phones, baby monitors, and some smart home devices can use 800 MHz–1.9 GHz bands. High-power appliances, in particular, produce stronger fields that may interfere with nearby electronics and contribute to overall electromagnetic pollution in the home [36].

### 2.2. EMI Evolution in Time

**Figure 1:**
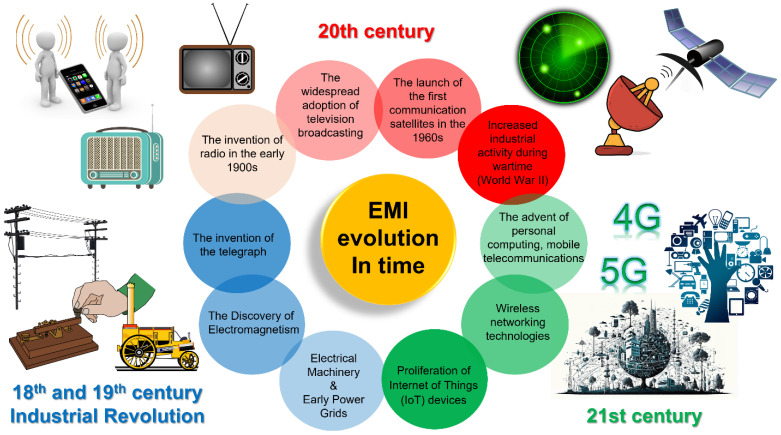
Evolution of the EMW emitters through the centuries.

The progress of technology has resulted in a significant increase in human-made sources of EMW pollution over the centuries (Figure 1). The Industrial Revolution, which unfolded during the late 18th and early 19th centuries, represents a defining epoch in human history—not only for its profound societal transformations but also for initiating substantial anthropogenic contributions to electromagnetic wave (EMW) pollution. This period of accelerated technological and industrial progress established the foundation of today’s complex electromagnetic landscape. Its most consequential advancements were the development and widespread implementation of electricity generation and distribution systems. Key developments during this period include: Foundational Discoveries in Electromagnetism with pioneering work by scientists such as Michael Faraday and Hans Christian Ørsted, which led to the identification of electromagnetic principles, forming the scientific basis for practical electrical technologies. Introduction of Early Power Grids, like the creation of electrical power distribution systems to meet growing industrial and urban energy demands, resulted in widespread exposure to extremely low-frequency (ELF) electromagnetic fields. Production of Electrical Machinery, with the use of components such as dynamos, electric motors, and transformers, generated electromagnetic radiation as an operational byproduct, contributing to localized sources of EMW pollution. The widespread integration of electricity, along with the establishment of power generation and transmission infrastructure, marked the onset of anthropogenic electromagnetic wave (EMW) sources. Early electrical networks and devices generated low-frequency electromagnetic fields, laying the groundwork for human-induced EMW emissions.

The 20th century witnessed unparalleled technological advancement, leading to a substantial increase in electromagnetic wave (EMW) pollution [37]. Progress in communication technologies, industrial development, and global infrastructure contributed to the widespread generation of human-made EMW emissions across diverse frequency spectra, significantly transforming the Earth’s electromagnetic landscape. The development of radio in the early 20th century, followed by the widespread acceptance of television broadcasting by the mid-century, and the launch of the first communication satellites in the 1960s, introduced high-frequency electromagnetic waves. These innovations dramatically boosted the density of EMWs, extending electromagnetic pollution beyond the Earth’s surface. Heightened industrial activity during wartime—particularly during World War II—significantly intensified EMW emissions, driven by the increased reliance on technologies such as radar and radio communication. Radar systems, developed during the war, introduced high-power microwave emissions. In the post-war era, radar applications expanded into civilian sectors, including meteorology and air traffic control, further contributing to EMW proliferation. Additionally, mid-20th-century nuclear weapons testing generated intense electromagnetic pulses (EMPs), which disrupted communication infrastructure and underscored the destructive capabilities of EMWs. The late 20th century observed the widespread emergence of personal computers, video screens, domestic electronics, and mobile phone service, bringing the ubiquitous RF emissions and contribution to localized EMW pollution.

The early 21st century has witnessed an unprecedented escalation in electromagnetic wave (EMW) pollution, largely driven by the digital revolution [38]. The widespread adoption of personal computing, mobile communication, and wireless networks has led to an exponential increase in electromagnetic emissions. The deployment of 4G and 5G networks, alongside the rapid expansion of IoT devices, has further intensified EMW exposure by introducing a higher frequency of EMWs and increasing the density of wave propagation in the modern electromagnetic environment. Emerging technologies—including renewable energy systems, autonomous vehicles, and smart city infrastructures—mark the latest stage in the progression of EMW pollution. As the EMW exposure grows increasingly congested, the demand for sustainable strategies and regulatory frameworks has become more urgent than ever.

## 3. EMI Effect and Concerns

A medical device refers to any instrument, apparatus, machine, material, or related product—including essential software—intended by the manufacturer for use on humans. It is used for medical purposes such as diagnosis, treatment, or surgery. Unlike pharmaceutical drugs, which exert biochemical effects, medical devices primarily act through physical means on the human body. The growing incorporation of electronic components in medical devices has made their reliability and safety increasingly vulnerable to EMI. Contemporary medical equipment—such as pacemakers, ventilators, infusion pumps, imaging systems, and wearable health monitors—depends on electronic circuits and wireless communication for precise operation. However, exposure to various EMW emitters, including mobile phones, radio transmitters, power lines, and MRI machines, can cause interference. This EMI may lead to data inaccuracies, signal distortion, or, in some cases, device malfunction.

### 3.1. EMI Effect on Medical Devices

Effects of EMI on Medical Devices can be classified as:

1. Functional Disruption:
  - EMI can impair the performance of critical devices such as pacemakers and defibrillators, potentially causing irregular operation or failure to deliver essential therapy.
  - Diagnostic tools like electrocardiograms (ECGs) and electroencephalograms (EEGs) may produce distorted signals, compromising the accuracy of medical evaluations.
2. Safety Hazards:
  - Interference with devices like ventilators or infusion pumps may lead to incorrect delivery of oxygen or medication, posing serious risks to patient health.
  - Communication issues in wireless telemedicine tools can disrupt real-time monitoring and treatment.
3. Reduced Device Lifespan:
  - Continuous EMI exposure can gradually degrade electronic components, diminishing the reliability and operational life of medical equipment.

Literature reports highlight various effects of radio transmitters on medical device performance [39]. The GSM mobile network, in particular, is a known source of EMWs, with documented cases of interference affecting devices such as pacemakers, implantable cardioverter-defibrillators (ICDs) and automated external defibrillators (AEDs), cardiac monitors, infusion pumps, and ventilators [40,41]. Bassen et al. [42] conducted similar research, finding that EMI occurred only when mobile phones were placed 2.3–5.8 cm from the ICD pulse generator, which was partially submerged in saline. Removing the phones eliminated interference. Three ICD models were tested using two digital and one analog phone, all operating at full power. EMI varied among devices: one ICD showed pacing inhibition, while the others experienced unintended high-voltage discharges when exposed to a time division multiple access (TDMA)-11 Hz phone at close range. The most sensitive ICD reacted up to 5.8 cm away. A TDMA-50 Hz phone also caused inhibition and activation at 2.3 cm. EMI was strongest when the phone’s antenna was aligned above the ICD header. In contrast, testing a 900 MHz phone on an AED showed no detectable interference on the ECG display [43]. Censi et al. [44] explored how global system for mobile communications (GSM) radio frequency (RF) signals from mobile phones can interfere with cardiac pacemaker (PM) function. Their review showed that older pacemakers are more prone to EMI than newer models, largely because modern devices include RF feedthrough filters that enhance shielding. In earlier PMs lacking these filters, internal nonlinear components may partially demodulate modulated RF signals. This can be problematic, as digital phones use very low-frequency modulation (as low as 2 Hz), which may be misinterpreted as normal heart activity. Feedthrough filters help block RF signals from entering the device, effectively reducing EMI across a broad frequency range. Buczkowski et al. [45] investigated the impact of mobile phones as a source of EMI on the reliability of medical devices, specifically focusing on ECG systems. Their study showed that interference with ECG electrodes depended on the GSM antenna’s design and placement, as well as the construction of electrode leads and amplification circuits. Even inactive phones periodically emit low-frequency bursts, which can introduce artifacts resembling fibrillation, especially in discontinuous transmission (DTX) mode. To avoid such disruptions, they recommended keeping mobile phones at least 7.5 cm away from ECG electrodes during recordings. emphasized the need for further research to assess GSM-related EMI across different medical devices, particularly those using automated algorithms like AEDs. Baranchuk et al. [39] examined the effects of EMI from various communication devices—including GSM and CDMA phones, analog phones, Wi-Fi, and pagers—on ECG machine performance. EMI was tested on three ECG models (MAC 5000, MAC 1200, ELI 100) at distances ranging from 2 m to direct contact with the acquisition module. Interference was observed only on the MAC 5000 when phones were placed directly on the module. No EMI occurred with other devices or at greater distances. ECG readings were evaluated by a diverse group of medical professionals to assess interpretation errors caused by EMI. Misinterpretation occurred in 18% of cases, commonly mistaken for atrial fibrillation/flutter (52%), ventricular arrhythmias (22%), or pacemaker malfunction (26%). Medical students and non-cardiology residents showed significantly lower accuracy in identifying EMI effects. Van Lieshout et al. [46] studied the impact of new-generation mobile phones (2G operating at 850 to 1900 MHz, and 3G operating at 850 to 2100 MHz), on 61 types of medical devices by testing GPRS (900 MHz, 2 W) and UMTS (1,947.2 MHz, 0.2 W) signals under controlled conditions. EMI events were classified using a critical care event scale. Out of 61 devices tested across 17 categories, 26 devices (43%) experienced 48 EMI incidents, including 16 dangerous, 20 serious, and 12 minor cases. The GPRS-1 signal caused the most interference (41%), followed by GPRS-2 (25%), while UMTS caused the least (13%). EMI typically occurred within a median distance of 3 cm from the device, with some hazardous events recorded as far as 300 cm away. The authors recommended maintaining a 1 m distance between mobile phones and critical care equipment to minimize interference while allowing convenient phone use nearby. Wallin et al. [47] evaluated the electromagnetic compatibility of GPRS, UMTS, and WLAN with 76 medical devices in intensive care and operating rooms, including clinical observations during 11 surgeries and 100 h of ICU care. UMTS and WLAN signals produced minimal interference, indicating safe use in critical care settings. However, GPRS signals caused an infusion pump to stop at 50 cm. Their findings support the safe use of GPRS devices at a distance of 1 m, with minimal interference risk in public areas. Van der Togt et al. [48] examined RFID-induced EMI on 41 critical care medical devices using active 125 kHz and passive 868 MHz RFID systems. Across 123 tests, 34 EMI events were recorded, with 22 classified as dangerous. The passive 868 MHz RFID caused more frequent interference (63%) than the active 125 kHz system (20%). The median distance for EMI was 30 cm, ranging up to 600 cm. The study concluded that RFID can trigger potentially dangerous EMI in medical devices, highlighting the need for on-site EMI testing and updates to international standards before deploying RFID in critical care environments. Together, these studies emphasize the susceptibility of medical equipment to EMI from wireless technologies and underscore the importance of maintaining safe distances, rigorous testing, and regulatory updates to ensure patient safety.

Gwechenberger et al. [49] investigated whether a commercially available pulsed electromagnetic field (PEMF) therapy system, intended for unsupervised clinical and home use, could cause EMI with implanted pacemakers (PMs) and automatic implantable cardioverter defibrillators (AICDs). Fifteen PMs and five AICDs were tested using a torso phantom and exposed to magnetic fields from three applicators: a full-body mat, a cushion, and a bar. The study concluded that bipolar electrode setups prevent EMI from the PEMF system, whereas unipolar settings in pacemakers carry potential risks. Tiikkaja et al. [50] conducted an in vivo study on EMI susceptibility in 11 pacemaker and 13 ICD users. Participants were exposed to magnetic fields (2–200 Hz, up to 300 µT) using a Helmholtz coil and to fields from common sources like electronic article surveillance (EAS) gates, induction hobs, and MIG welding tools. All pacemakers were tested in bipolar mode, with three also evaluated in unipolar mode. None of the bipolar-configured devices exhibited interference. However, unipolar PMs were affected by the strongest Helmholtz coil fields, and one also showed interference from the EAS gate and welding equipment. The induction hob had no effect. However, unipolar configurations present a higher risk in environments with strong electromagnetic fields and should be avoided when possible. Overall, both studies emphasize the greater EMI resistance of bipolar settings and highlight the vulnerability of unipolar pacemakers when exposed to strong or localized magnetic fields.

Silny et al. [51] conducted extensive lab tests on over 100 pacemakers, both old (produced in XZ) and modern, to explore worst-case EMI scenarios involving low-frequency fields. Real-world simulations, including exposure to high-voltage power lines and electronic surveillance systems, are ongoing. Early findings showed that a unipolar, ventricularly controlled pacemaker implanted in the left chest could experience interference at electric field strengths above 5 kV/m. For example, a 50 Hz vertical electric field of 1 kV/m could induce about 400 µVpp at the pacemaker input. Magnetic fields also induce interference by generating voltages through multiple induction loops formed by the body and pacemaker system, with effective loop areas ranging from 100 to 221 cm^2^. In unfavorable setups (e.g., left-pectoral, atrially controlled), interference thresholds ranged from 16 to 552 µT at frequencies between 10 and 250 Hz. While such interference can occur in daily life, studies suggest the actual risk is low. This is likely due to a narrow sensitivity band in most pacemakers, making them resistant under normal conditions despite extreme laboratory results.

When exposed to EMI, cardiac pacemakers may enter an inhibitory mode or initiate rapid asynchronous pacing [52]. Stronger interference can cause continuous asynchronous pacing, potentially posing serious risks. A pacemaker’s susceptibility to EMI depends on factors such as its electromagnetic shielding, calibration, type (unipolar or bipolar), anatomical placement, the patient’s body size and position in the field, and physiological factors like breathing. The characteristics of the interfering field—whether electric, magnetic, or both—as well as its frequency and modulation, also influence the device’s response.

In summary, although cardiac pacemakers may be affected by low-frequency EMI in specific setups, the real-world risk remains minimal for most patients. On the other hand, contemporary electrocardiogram (ECG) devices can be disrupted by EMI from sources like analog/digital phones and power lines, particularly when these are in close proximity to the ECG acquisition module.

### 3.2. EMI Effect on Electronic Devices

Over the past decade, the use of electronics and their components has steadily increased, making them more vulnerable to the effects of EMWs. These effects vary based on the type, strength, and frequency of the EMW and can significantly impact device performance [30]:

1. EMI occurs when external EMWs disrupt the normal function of electronic systems. Sources include radio transmissions, cell phones, power lines, microwaves, and industrial equipment. Effects range from communication errors and signal distortion to the malfunctioning of sensitive devices and degraded wireless network performance. Mitigation involves shielding (e.g., Faraday cages), proper grounding, and signal filtering [53,54].
2. Electromagnetic Pulses (EMPs) are high-intensity EM bursts from natural sources like lightning and solar flares or artificial events such as nuclear explosions. These pulses can overload circuits, cause data loss, and permanently damage devices. Protection methods include hardened circuits, surge protectors, and shielded enclosures [55].
3. Radiofrequency (RF) Interference, the RF waves, used in mobile communications and radar, can overheat components and interfere with devices like pacemakers. Ensuring RF compliance and incorporating safety standards in design helps prevent such issues [56,57].
4. Electrostatic Discharge (ESD) is a sudden current flow caused by contact or a strong EM field, often damaging sensitive components. ESD protection is vital in device manufacturing and handling [58,59].
5. Chronic Exposure to Low-Intensity EMWs, such as those from nearby power lines, may slowly degrade electronic parts like resistors, capacitors, or semiconductors [60].

Overall, external EM fields pose a real risk to electronics by generating disruptive signals within systems. Proactive design and protective measures are essential to ensure safe and reliable device operation.

EMP pulses pose a significant threat to electronic systems, either intentionally or through exposure to ambient high-frequency signals, often in the microwave range. Such interference can severely damage or destroy semiconductor components, leading to potentially catastrophic system failures. EMI can disrupt communication signals by:

- Adding noise, which obscures the intended signal,
- Weakening signal strength (attenuation),
- Causing frequency shifts that disrupt synchronization.

Already distorted signals are even more vulnerable to EMI due to lower signal integrity, higher error rates, or nonlinear interactions that produce further distortion.

EMI can affect mobile devices, Wi-Fi, Bluetooth, Ethernet, DSL, coaxial lines, and satellite communications—resulting in dropped calls, slow data speeds, or poor GPS accuracy. Nearby electronic equipment may induce noise into connected systems, degrading performance. Mitigation strategies include using high-quality, certified electronics, employing error correction, filters, and shielded or twisted-pair cabling, replacing copper with fiber optic cables, which are immune to EMI, and designing wireless networks with careful attention to transmission power, site placement, and proximity to high-voltage sources. In high-speed networks, EMI management is essential to maintain reliable and interference-free communication [30].

The effects of EMI vary depending on the device type and its operational mode [53]. To reduce EMI impact on distorted signals, communication systems should implement:

- Shielding: Enclose sensitive parts in metal to block EMI.
- Filtering: Remove unwanted frequencies and reduce noise.
- Error Correction: Use algorithms to recover signal integrity.
- Grounding: Properly ground devices to lower interference.
- Spread Spectrum: Apply techniques like frequency hopping to avoid consistent EMI exposure.

Standard EMI compliance tests use a line impedance stabilization network (LISN), which only provides a pass/fail result for total conducted noise, without distinguishing between common mode (CM) and differential mode (DM) interference. This lack of detail makes EMI filter design difficult. While some networks aim to separate CM and DM noise, they often perform poorly at high frequencies due to parasitic effects and are costly and complex. To address this, Xiao-hui et al. proposed a new method using independent component analysis (ICA) and signal statistics to detect EMI in underground power electronics. Their technique offers a simple, robust, and cost-effective solution for EMI detection [61].

Yang et al. proposed a synthetic system for automated analysis and suppression of conducted EMI noise [54]. It collects EMI signals from the equipment under test (EUT) using a line impedance stabilization network (LISN), separates them into common mode (CM) and differential mode (DM) via a noise separation network, and processes the signals through FPGA-controlled amplification and filtering (10 kHz–30 MHz). The data is then digitized and sent to a computer for analysis and suppression planning. This system has proven effective in both technical research and applications. Muttaqi et al. studied EMI caused by fast-switching power devices (e.g., IGBTs) in converters and industrial equipment [62]. Their work explored EMI generation mechanisms, coupling paths, and impacts, including health risks and equipment failure. They developed an EMI measurement system for time- and frequency-domain analysis and proposed filtering methods to reduce EMI in PWM IGBT-inverter motor drives.

The growing reliance on electronic systems has heightened their vulnerability to various electromagnetic disturbances, including EMI, EMP, RF interference, ESD, and chronic low-intensity exposure. These phenomena can disrupt communication, degrade performance, or cause irreversible device failure. Effective mitigation requires a combination of shielding, grounding, filtering, error correction, and compliance with EMI standards. Advanced diagnostic and suppression methods, such as ICA-based detection and automated noise separation systems, offer promising solutions for managing interference in modern high-speed and power electronic applications.

### 3.3. EMI Effect on Humans

Before the Second World War, the health effects of EM radiation were neglected [63]. Later, cases of sleep disturbance, headache, and fatigue were reported; the EMF influence on human health was considered [64]. Preliminary studies indicated negative biological effects of EMF, such as circadian rhythm alteration, hormonal imbalance, changes in intracellular ion levels, effects on the memory center, blood–brain barrier (BBB) change, and even brain tumors [65]. Several organizations have drawn contradictory opinions on this topic. Naley, in 2011, the International Agency for Research on Cancer labeled ELF- and RF-EMF as possibly carcinogenic for humans [66]. Afterward, summarizing the results obtained in vitro and by the meta-analyses, the World Health Organization (WHO) and the Scientific Committee on Health, Environmental and Emerging Risks (SCHEER) of the European Union emphasized the unreliability of previous research, the lack of evidence, and conflicting results. Hence, the final opinion has not yet been formed [6].

Due to its nature, EMF can interact with the electrophysiological activities of the human organism. The most vulnerable systems to external EM radiation are the cardiac and the central nervous system (CNS), owing to the presence of electrically charged particles and the delicate nature of their biological processes. Considering that the heart and brain are composed of 73% water, when exposed to EMF, water molecules can be energized or polarized, leading to cell and tissue disruption [67,68]. Researchers indicated the impact of EMF on heart rate variability (HRV)—a measure of the variation in time between heartbeats. Specifically, Misek et al. reported that a real RF-EMF signal from the base station at 1805–1870 MHz frequencies led to an enhancement in HRV parameters [69]. Ubed et al. demonstrated that mobile phone EMF can affect HRV parameters, where the effects are stronger when subjects are chronically exposed and when the source is closer to the heart [70]. Other studies highlighted that EMF can interfere with active implanted devices (AIMD) such as pacemakers or implantable defibrillators. The case study conducted by Mattei et al. showed that the presence of the radio-frequency identification (RFID) readers (125 MHz–960 MHz) and Wi-Fi (2.4 GHz, transmission power is higher than 120 W) near patients with AIMD carries a risk of device damage [71]. Albanna et al. pointed out that a very low frequency-electromagnetic field (VLF-EMF) at the frequency of 10 kHz may enter the implant can and induce a voltage directly in the electric circuit, causing a malfunction of the electronic components [7]. Also, they noticed that during the exposure to RF-EMF, the electrode of AIMD acts as an antenna, in which EMF can induce a voltage and disturb the sensing ability of the device. On the contrary, Martinelli et al. demonstrated that short-term (48–72 h) and long-term (14 days) exposure to EMF of 915 MHz did not significantly affect myocardial, apoptotic, metabolic, and fibro-inflammatory profiles in mice [72].

**Figure 2:**
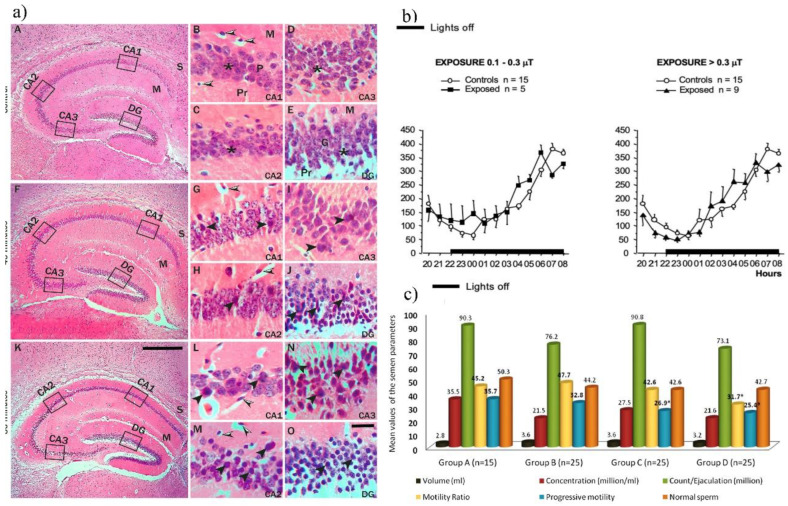
(**a**) Hematoxylin and Eosin-stained sections of the hippocampus of control (**A**–**E**), 40 min (**F**–**J**), and 60 min (**K**–**O**) exposed mice formed by Cornu Ammonis (CA) as CA1, CA2 & CA3 areas [74]; (**b**) Cortisol secretory patterns in 14 healthy male electricity workers, working in an extra high voltage station with a history of long-term exposure (1–20 years) to extremely low frequency electromagnetic fields (ELF-EMFs) [80]; (**c**) Basic parameters of semen samples in the study groups. *P < 0.05 [81].

Regarding EMF’s impact on the nervous system, researchers have found that its effects are noticeable in the hippocampus. It has been shown that EMF in the frequency range of 50 kHz to 2400 MHz influences working memory during long-term exposure (7 weeks) and causes anxiogenic effects, enhances memory, induces oxidative stress, and alters the microglia cell population in the mice’s hippocampus after 2 months of exposure [73]. Additionally, a reduction in pyramidal cell numbers in the mice’s hippocampus and anxiety-like behavior has been observed after 60 min [74] (Figure 2a) and 21 days [75]. Other studies indicate that RF-EMF sources operating between 1 and 2.45 GHz result in oxidative stress and mitochondrial imbalance in neuronal-like cells after 48 h, as well as apoptosis, and cholinergic dysfunction in the mouse hippocampus and cerebral cortex after 4 to 8 weeks [76].

The EMF can interfere with hormone production, release, and reception [77]. The study conducted on human growth hormone showed that mobile phone radiation (940 MHz) led to a change in the hormones’ secondary structure during 45 min of exposure [78]. Also, a 27% in protein size as well as a lifetime decrease were noticed. Human circadian rhythm (CR) may be affected by EMF. Changes in levels of melatonin and cortisol in different parts of the day regulate CR. A recent investigation conducted by Selmaoui et al. showed that individuals who were chronically exposed to ELF-EMF for 5 years had significant changes in melatonin [79] and cortisol [80] levels (Figure 2b) in human blood and serum, respectively.

The male reproductive system has proven to be extremely sensitive to EM radiation due to its sensitivity to environmental changes, and because mobile phones and wireless-based personal computers are usually worn near reproductive organs [82]. Testicles are especially damaged through oxidative stress and heat conducted by RF-EMF from devices [83]. Some analyses have determined that RF-EMF caused a degeneration in testicular tissue, a decline in testosterone levels [84], a decrease in sperm quality and motility in men between 25 and 50 years [81].

Given that 90% of all work today requires working on a computer, attention to the EMF impact on the ocular system must be paid [85]. The fact that 96% of young people aged 16–29 years in the EU use the internet every day, while 69% of 9- to 22-year-olds use social media or play games more than 3 h a day [86], indicates the seriousness of this problem. Several case studies showed that RF-EMF from mobile phones caused defects in refraction for both eyes in 15- to 18-year-olds [87], asthenopia symptoms (19- to 25-year-olds volunteers) [88], dry eye symptoms (12- to 26-year-old adolescents) [89]. An experimental study conducted by Shokoohi-Rad et al. showed that mobile phone radiation increased intraocular pressure in patients between 40 and 70 years who suffered from glaucoma [90]. In an in vitro study, Oladnabi et al. established that ELF-EMF of 50 kHz has not been able to induce multiplication and death of pigment cells in the retina, but can change the expression of proangiogenic genes, causing neovascularization.

It can be assumed that EMF at different frequencies mostly targets electrosensitive systems such as the cardiac and nervous systems. Then, the impact on hormones that control critical metabolic processes, as well as the day/night rhythm, can be observed. Except that the highly vulnerable organs are those that are close to the EMW emitter.

### 3.4. EMI Effects on the Environment

Until now, no legislation has taken the effects of EMF on the environment into consideration. As stated in the International Commission of Non-Ionizing Radiation Protection (ICNRP 2000) regulation, the impact of EMF on non-human species is equated with the impacts on humans [91]. Existing criteria for the impact on humans cannot be applied to animals or plants due to their different anatomy, physiology, reception structures, environmental conditions, distance from the radiation source, etc. Flying birds, insects, and tall woody plants are often found near overhead cables for the electricity supply. Besides, aquatic animals and plants are constantly exposed to radiation arising from undersea cables [92].

**Figure 3:**
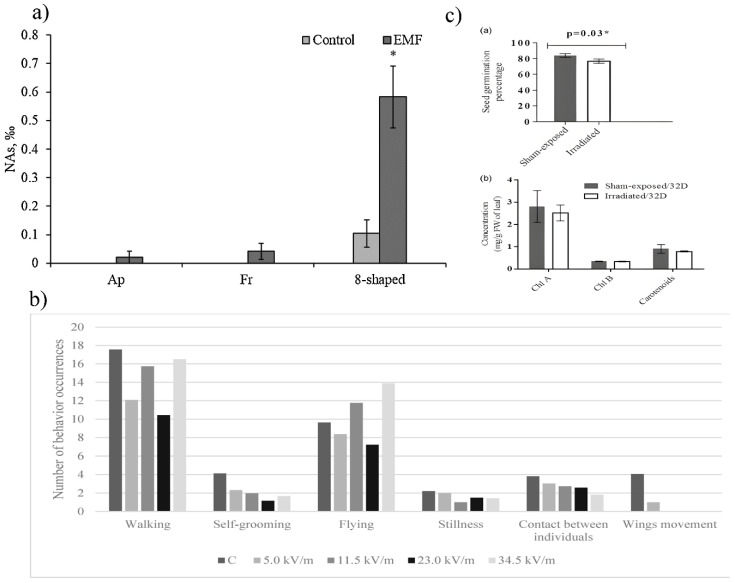
(**a**) Cytotoxicity endpoints (NAs: apoptotic (Ap), fragmented (Fr), and 8-shaped nucleus erythrocytes) induced in *O. mykiss larvae* (22–26 mm TL) after exposure to EMF of 1 mT for a period of 40 days [113]; (**b**) Average time spent on behavior by bees after 12 h under the influence of an E-field at 50 Hz [100]; (**c**) Effect of electromagnetic (EM) irradiation on seed germination rate and photosynthetic pigment concentrations [4]. * Statisticaly important results.

Numerous studies examined the effects of EMF emitted from sub-sea cables on marine animals. Dunham et al. monitored the population dynamics of deep-dwelling glass sponges settled around the cable during 4 years [93]. It was observed that the number of living individuals decreased by 55% within 3.5 years. Investigations conducted on the mollusk *Onchidium struma* [94] and crustaceans *Cancer pagurus* [95] and *Homarus gammarus* [96] showed that EMF from underwater cables triggered immune response and stress-related mechanisms in these species. It has been revealed that underwater cables operate at the frequency of 50 Hz [97]. It was observed that this ENW emitter led to the yolk sac absorption rate and caused nuclear abnormalities in *Oncorhynchus mykiss* larvae after 36 and 40 days (Figure 3a) of exposure, respectively [76]. Electrosensitive groups of fish, such as elasmobranchs, are especially vulnerable to EMF [98]. They use EMF for localization, spatial orientation, and intraspecies interaction, so their behavior can be affected by artificial EMF sources. By observing the behavior of two elasmobranch species, *Beringraja binoculata* and *Caliraja rhina*, during exposure to subsea cable EMF (60 Hz), Newton et al. noticed higher activity and changes in habitat usage, speed of movement, and body angle in *B. binoculata* [99]. On the contrary, reduced activity of *C. rhina* individuals was found.

A significant issue related to the EMF impact on the environment is the effects on pollinating species such as the honey bee (*Apis mellifera*) [79]. In a series of successive studies, Migdal et al. demonstrated that EMF at the frequency of ELF-EMF (50 Hz) altered major metabolic pathways (β-oxidation, citric acid cycle, and ATP production) in honey bees [100]. As a consequence, activities such as flying, walking, self-grooming, stillness, and wing movements were changed (Figure 3b). Except for ELF-EMF, honey bees can be affected by RF-EMF. The same research group showed that RF-EMF at 900 MHz led to an increase in urea level in *A. mellifera*’s haemolymph during 1–3 h of exposure [101]. Also, alanine- and aspartate aminotransferase (ALT and AST) levels were reduced, causing suppression in protein synthesis in which they are involved. Odemer et al. revealed that mobile phone radiation (900 MHz) reduced the hatching ratio of adult queens after 11 days of exposure [102]. Additionally, higher frequencies, even of 5.8 GHz, can affect *A. mellifera*’s navigation prowess [103]. Apart from honey bees, EMF influences other groups of insects. The EMF of 50 Hz is found to be a disruptive factor for calling song patterns in *Gryllus maculatus* [104]. In another study, it has been shown that mobile phone radiation of 900 MHz can change the distribution of insects such as *Ixodes ricinus*, well-known in human medicine as a vector for *Borrelia* spp. and *Rickettsia* spp. pathogens transmission [105]. As *I. ricinus* uses EMF to locate the host, it is considered that EMF from electrical devices could even more attract these insects in urban areas. This may increase the risk of Lyme disease.

EMF can influence magnetoreception in species, disrupting their ability to navigate using the Earth’s magnetic field. Numerous studies showed that ELF-EMF of 50 Hz can interact with bio magnetite (Fe_3_O_4_) in the roundworm *Caenorhabditis elegans* and alter their orientation and communication [106].

However, the greatest impact is observed on migratory birds. It was suggested that RF-EMF, particularly in the MHz range, can affect the radical pair magnetic compass by disrupting a spin state. It altered birds’ ability to orient themselves using natural magnetic fields, as well as impaired their migrations [107]. Thus, the RF-EMF of 7 MHz led to disorientation of the European robin (*Erithacus rubecula*) [108], and RF-EMFs between 75 and 80 MHz disrupt the magnetic compass of the Eurasian blackcaps (*Sylvia atricapilla*) [109]. It was found that RF-EMF, ranging between 120 and 220 MHz, interacts with the flavin/tryptophan radical pair in cryptochrome and disorients *S. atricapilla* [110].

The effects of EMFs are especially obvious on animal groups that are highly sensitive to changing environmental conditions (stenovalent), such as amphibians. Boga et al. demonstrated that RF-EMF (900 MHz) caused changes in body growth in male and female as well as aggressive offspring behavior of *Xenopus laevis* [111]. In another study, it was shown that RF-EMF of 22 MHz enhanced growth, and at the same time, mortality of *Rana temporaria* tadpoles [112].

The effects of EMF on plants are easy to monitor due to their immobility, constant orientation to EMF, and high surface area to volume ratio, which makes them more susceptible to interacting with EMF. Also, the formation of genetically stable lines through asexual reproduction and self-pollination, and metabolic mutants, allows a better understanding of the way of EMI signal transduction. Plants detect EMF signals and transduce them into molecular responses. Generally, EMF effects on plants are as follows: alteration of enzymes, especially those involved in reactive oxidative species (ROS) metabolism, changes in gene expression, and modification of plant development [114]. According to the previous research, RF-EMF is usually associated with oxidative stress in plants. Sharma et al. demonstrated that after 4–8 h of exposure to RF-EMF (1800 MHz), levels of antioxidative enzymes such as guaiacol peroxidase, glutathione-S-transferase, ascorbate peroxidase, catalase, glutathione reductase, and superoxide dismutase increased in *Trigonella foenum-graecum* seeds [115]. Alike effects are observed in *Brassica oleracea* cultivars after one day of exposure to EMF at the frequency of 2850 MHz [5]. Otherwise, ELF-EMF can interact with the stress mechanism, making the response more pronounced. It was noted that the ELF-EMF of 14.3 Hz increased the resistance of wheat (*Triticum aestivum* L.) to drought [116]. Plant germination can also be modified by different EMF frequencies. Thus, the RF-EMF of 1837.50 MHz emitted from Wi-Fi reduced the germination rate and photosynthetic pigment concentration of rice var. *Satabdi* seeds (Figure 3c) [4], while ELF-EMF (10 Hz) improved germination in Foxtail millet (*Setaria italica*) seeds [117]. Also, EMF can influence plant growth. Surducan et al. proved that RF-EMF of 915 MHz led to an increase in height of exposed plants and root, stem, and leaves dry mass, as well.

Summarizing effects of EMF on animals, it can be concluded that the most damaged are electro- and magneto-sensitive species, populations of animals whose habitats are near EMF sources, stenovalent species, as well as those that play critical roles in ecosystems. As far as plants are concerned, it can be noticed that negative or positive EMF effects depend on the radiation frequency. The impact of EMF on the environment is more than obvious, so establishing special regulations that limit EMFs in the environment is urgently needed, as in the case of humans.

## 4. EMI Market and Forecast

**Figure 4:**
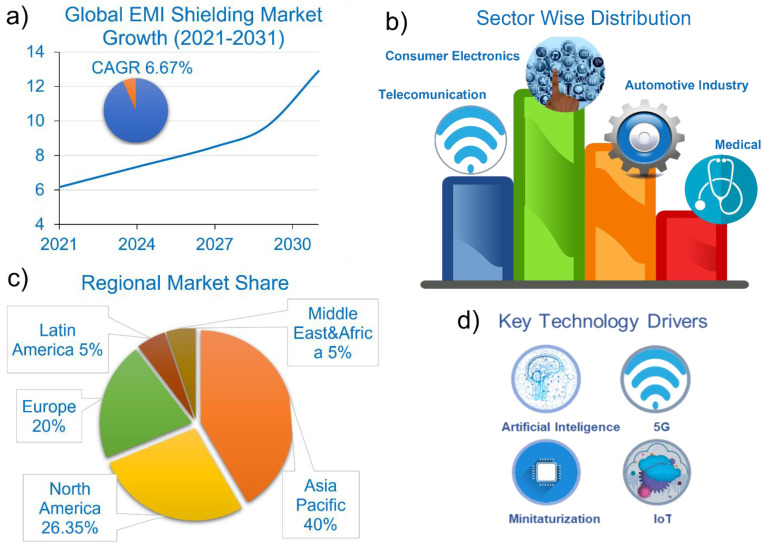
EMI market trends express the global growth by sector (**a**), global EMI market growth, (**b**) sector–wise distribution, (**c**) regional market, and (**d**) key technology drivers.

A major limitation of existing literature on EMF shielding is the lack of peer-reviewed studies. Most claims are published in non-scientific outlets, such as bulletins or online platforms, due to the absence of rigorous, scientifically validated research confirming the effectiveness of shielding in reducing EMI-related symptoms. This type of investigation remains difficult to conduct reliably, especially since the EMI shielding market is undergoing substantial expansion, fueled by the widespread adoption of electronic devices across multiple sectors (Figure 4).

Valued at around USD 7.34 billion in 2024, the market is projected to grow to USD 9.69 billion by 2029 and reach USD 12.9 billion by 2031, representing a compound annual growth rate (CAGR) of 6.9% between 2024 and 2031 [118,119].

The global EMI shielding market is being driven by several critical technological advancements and increasing industry demands:

- Proliferation of Electronic Devices
- Advancements in Wireless Technologies
- Miniaturization of Electronic Components
- Impact of Artificial Intelligence (AI)

The widespread adoption of electronics across consumer products, automotive systems, telecommunications, medical devices, and industrial machinery has substantially heightened the demand for effective EMI shielding solutions to ensure optimal device performance, operational stability, and regulatory compliance. In 2021, the consumer electronics sector dominated the global EMI shielding market, holding a 33.1% market share. This segment is projected to grow at a moderate compound annual growth rate (CAGR) of 6.67% during the forecast period [119].

The ongoing deployment of 6G infrastructure and the expansion of IoT-connected devices have intensified EMI challenges, necessitating the development and integration of advanced shielding technologies capable of preserving signal integrity and device functionality. Telecommunication towers are vital for communication systems, supporting first responders, healthcare, and law enforcement. With ~20,000 new base stations added yearly and 400 million new mobile subscribers, rising rural penetration and device miniaturization demand stronger EMI shielding due to higher frequency vulnerability [120]. This trend is accelerating the demand for high-performance shielding materials compatible with compact and integrated device designs. The rise in the number of smartphone users—from 2.9 billion in 2018 to 3.5 billion in 2020—highlights this growing demand [119].

Artificial intelligence (AI) has also emerged as a transformative force across multiple industries. AI-enabled technologies, such as autonomous vehicles, industrial automation, and smart appliances, involve high-frequency data transmission and advanced processing systems that are particularly sensitive to electromagnetic interference. Efficient EMI shielding is therefore critical to ensure uninterrupted operation and prevent signal corruption in AI-based applications.

The EMI shielding market encompasses a variety of materials, including coatings and paints, conductive polymers, elastomers, metal-based shielding solutions, EMI/EMC filters, and EMI tapes and laminates. Each material offers unique advantages depending on application-specific requirements, such as cost efficiency, mechanical flexibility, thermal stability, and shielding effectiveness [121].

The EMI shielding market shows clear regional segmentation driven by technological maturity, industrial activity, and regulatory frameworks. Asia-Pacific dominates with ~40% of global revenue (2023), supported by rapid industrialization, electronics manufacturing, and 5G deployment in China, Japan, and South Korea. North America (26.35% in 2022) benefits from aerospace, defense, and healthcare demand, with the U.S. contributing over 80% regionally. Europe (20% in 2023) is sustained by strong automotive/aerospace sectors and strict EMC regulations, led by Germany, France, and the UK. Latin America and the Middle East & Africa each hold ~5%, with growth driven by industrialization and telecom expansion market leadership [122].

In conclusion, the EMI shielding market is experiencing robust growth, underpinned by technological advancements, the rapid proliferation of electronic devices, and expanding industrial applications. The Asia-Pacific region continues to lead, driven by its strong manufacturing base and telecom expansion. North America and Europe maintain substantial shares, supported by innovation, regulatory mandates, and defense-related applications. Meanwhile, emerging markets in Latin America and the Middle East & Africa present promising opportunities for future investment and market expansion. As the global reliance on electronic systems deepens, the demand for innovative, high-efficiency shielding materials is projected to rise, offering substantial potential for technological and commercial development within this sector [9].

## 5. Prevention Against EMI

While it is impossible to entirely avoid exposure to electromagnetic waves (EMW), exposure can be minimized through the use of EMF-shielding materials, environmental and behavioral optimization, and regular inspection and maintenance practices.

Metal-based shielding products can reduce harmful anthropogenic EMFs, they may also block natural, non-ionizing EMFs essential to biological rhythms. Early studies from the 1960s–70s, involving participants in electromagnetically isolated environments, highlighted the importance of natural EMFs. Panagopoulos and Chrousos [22], advocated for urgent research into the safety and effectiveness of shielding methods when used alongside pulse-generating devices that replicate the frequency, amplitude, and waveform of natural atmospheric resonances.

**Figure 5:**
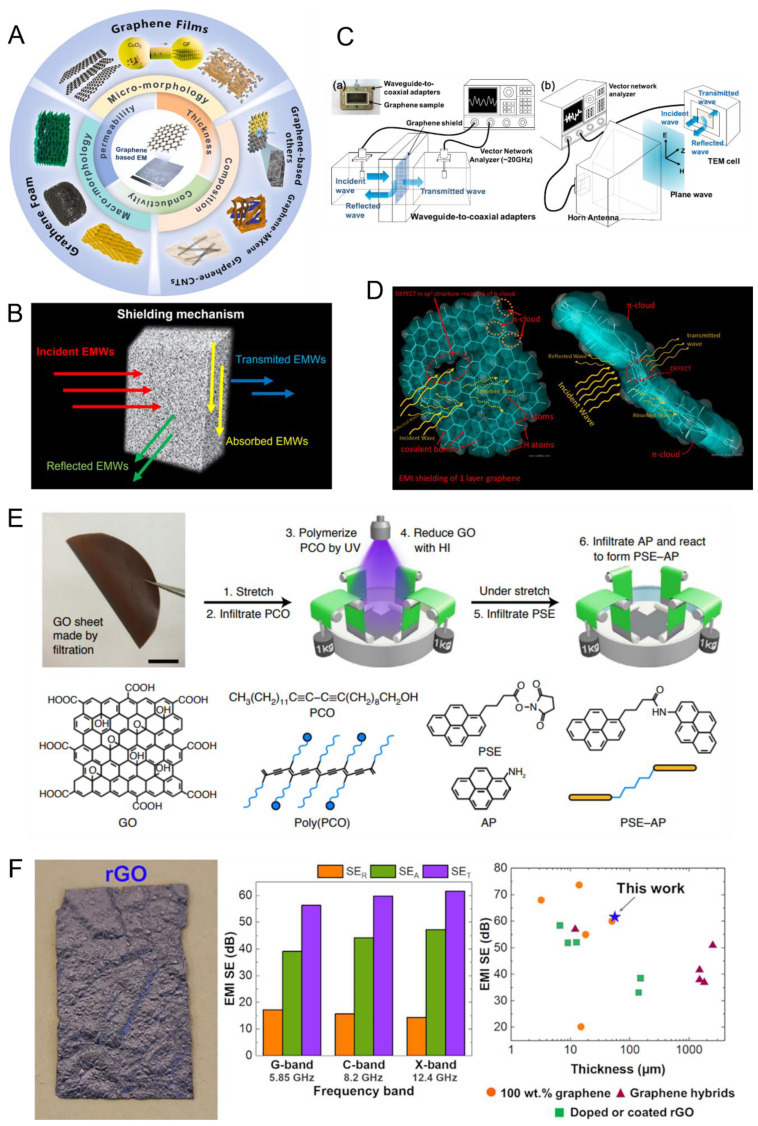
(**A**) presents the graphene-based materials for EMI shielding [14]; (**B**) represents the schematic illustration of the shielding mechanisms achieved through absorption, reflection, or scattering of electromagnetic radiation; (**C**) is an illustration of the experimental setup used to evaluate the EMI shielding effectiveness of graphene showing (**a**) Waveguide-based measurement system operating in the 2.2–7 GHz range, and (**b**) Experimental setup utilizing a horn antenna, a transverse electromagnetic (TEM) cell, and a vector network analyzer [134]; (**D**) illustrates the single-layer graphene structure, highlighting covalent bonds, π-cloud, and structural defects, along with the reflection, transmission, and absorption behavior of incident EM waves (top and side views) [12]; (**E**) illustrates the SB-BS-rGO sheets preparation [14]; (**F**) represents the image of the rGO free-standing film and EMI results [137].

EMW shielding materials play a vital role across various industries, including electronics, automotive, telecommunications, aerospace, and healthcare. Their primary function is to attenuate or block EMI, thereby preserving device performance and ensuring compliance with regulatory requirements. Depending on the application, shielding can be achieved through mechanisms such as absorption, reflection, or multiple reflection (Figure 5A), utilizing materials that range from traditional metals and conductive polymers to advanced nanocomposites.

Metals such as Cu, Ag, Ni, Al, and Fe remain the benchmark for EMI shielding owing to their high electrical conductivity, reflection-dominated shielding, well-established processing methods, and noticeable shielding effectiveness above 100 dB achievable. However, they also present significant drawbacks, including high density, susceptibility to corrosion and oxidation, challenges in fabrication of flexible or thin-film forms, high cost in certain cases (e.g., Ag, Cu), and limited environmental sustainability. Conductive polymers such as polyaniline and polypyrrole have been explored as alternatives, offering advantages including low weight, flexibility, corrosion resistance, and tunable conductivity. Still, their relatively low conductivity, thermal instability, and long-term degradation restrict their practical use (Table 1).

### 5.1. Carbon-Based Nanomaterials in EMI Shielding

Among the wide array of emerging nanomaterials, carbon-based nanomaterials [127,128,129,130,131] have garnered significant attention due to their exceptional multifunctional properties, such as:

- Mechanical robustness: Exhibiting high strength, flexibility, and elasticity.
- Chemical stability: Resistant to corrosive environments, including water, acidic, and alkaline conditions, with the ability to be chemically functionalized for tailored performance.
- Physical advantages: Possess low density combined with excellent mechanical strength.
- Electrical conductivity: Features high charge carrier mobility and tunable electronic band gaps.
- Processing versatility: Easily fabricated and incorporated into a variety of matrices and device architectures.
- Environmental compatibility: Biocompatible and non-toxic, supporting applications in both industrial and biomedical fields.
- Sustainability: Amenable to cost-effective, scalable production and recyclable processing.

Graphene-based materials, including graphene oxide (GO), reduced graphene oxide (rGO), carbon nanotubes (CNTs), carbon fibers, carbon black, and graphite, have exhibited outstanding potential for EMI shielding, with multi-layer, low-defect graphene achieving particularly high shielding effectiveness. Despite these promising properties, challenges related to scalability and cost-efficiency continue to hinder widespread industrial adoption. To advance the practical application of graphene in EMI shielding, future research should prioritize a balance of material quality with production yield through optimization of synthesis techniques, maintaining or improving shielding performance by development of lightweight, cost-effective composite systems, and/or promoting the large-scale graphene fabrication through implementation of sustainable and environmentally friendly approaches. With ongoing innovation, graphene and its derivatives are well-positioned to play a central role in next-generation EMI shielding technologies.

Graphene, due to its exceptional ballistic transport characteristics, can achieve electrical conductivities up to 3000 Scm^−1^ [132]. In addition to its electrical properties, graphene exhibits high thermal conductivity, along with corrosion resistance and excellent processability. Its compatibility with polymer matrices enables the fabrication of conductive composites, making it a promising candidate for use in thermal heaters, heat dissipation systems, thermal interface materials, and advanced EMI shielding technologies [133].

Hong et al. found that graphene monolayers produced by chemical vapor deposition (CVD) have a low EMI SE of 2.27 dB, primarily governed by absorption mechanisms [134]. The high sheet resistance of 635 Ω/sq indicated a significant presence of structural defects, which was correlated with poor shielding performance. In contrast, bilayer and tri-layer graphene samples demonstrated improved SE values of 4.13 dB and 6.91 dB, respectively, in the 2.2–7 GHz frequency range. As the number of graphene layers increased, a transition in the dominant shielding mechanism from absorption to reflection was observed—mirroring behavior commonly seen in thin metallic films, such as gold. The schematic representation of the setup is given in Figure 5B. Defect-free graphene structures have superior EMI shielding performance compared to their defective counterparts, with SE generally improving as the layer count increases [135]. Few-layer graphene produced using the same CVD technique, with a thickness of approximately 4 nm, achieved an SE of 19.1 dB in the 18–26.5 GHz range, maintaining a high optical transmittance of 80.5% [136]. Enhanced electrical conductivity in multilayered graphene samples contributed to increased reflection, further boosting EMI shielding efficiency compared to monolayer graphene. However, the practical limitations of CVD—including restricted scalability, technical complexity, and high production costs—render it less suitable for large-scale applications. As a result, bulk graphene materials for EMI shielding are more commonly synthesized via Hummer’s method, yielding GO, which is subsequently reduced to restore electrical conductivity. A schematic representation of single-layer graphene and its EMI shielding mechanism is provided in Figure 5C [12].

Extensive research has focused on the fabrication of free-standing graphene films through the layer-by-layer assembly of GO flakes, followed by chemical or alternative reduction methods aimed at eliminating oxygen-containing functional groups and restoring electrical conductivity [138,139]. Chemical doping is a powerful strategy to enhance the electrical conductivity of carbon nanomaterials by increasing charge carrier density and minimizing interlayer interactions [140,141,142]. In 2021, Wan et al. employed a method involving biaxial stretching of filter-fabricated GO sheets, which were sequentially modified through covalent bonding and π–π interactions (Figure 5E) [14,143]. These modified sheets were infiltrated with PCO and polymerized under UV irradiation. Subsequent reduction with hydrogen iodide (HI) facilitated the sequential infiltration and reaction of PSE and AP, forming PSE-AP molecules while preserving the biaxial alignment. The result was the production of high-strength, in-plane isotropic graphene sheets, exhibiting a tensile strength of 1547 ± 57 MPa, electrical conductivity of 1394 ± 65 S cm^−1^, and impressive EMI shielding effectiveness of 39 dB across the 0.3–18 GHz frequency range. Oliveira et al., reported outstanding EMI shielding performance of freestanding reduced (rGO) foil, fabricated through the thermal reduction in GO (Figure 5F) [137]. The resulting rGO foil, with an average thickness of 93.1 ± 12.4 μm, achieves a high shielding effectiveness (SE) of 61.6 dB at 12.4 GHz. This superior performance was primarily attributed to the thermal reduction process, which effectively restores the material’s electrically conductive network, yielding an electrical conductivity of 1.17 × 10^4^ Sm^−1^. To comprehensively evaluate the EMI shielding capabilities, measurements were conducted across the G- and C-bands, making this the first investigation to assess rGO films beyond the conventional X-band. The rGO foil consistently demonstrated EMI shielding efficiencies exceeding 99.999% across all tested frequency ranges, underscoring its strong potential for a broad spectrum of commercial applications requiring high-performance EMI shielding materials [137]. Milenkovic et al. synthesized GO and silver nanowire (AgNW) composites and investigated the strength and nature of the interactions between the two nanomaterials using density functional theory (DFT). The calculated interaction energy between pristine graphene and AgNWs was −48.9 kcal/mol, whereas the interaction energy between AgNWs and GO nearly doubled to −81.9 kcal/mol. These DFT results provided evidence of interfacial polarization at the GO–AgNW heterojunction, facilitated by charge transfer and accumulation at the interface, which enhances EMW shielding performance.

### 5.2. Advanced Carbon-Based EMI Shielding Materials

To improve EMI shielding performances of carbon-based nanomaterials, new forms of nanomaterials are produced, where foams attracted a large attention due to their mechanical strength, and EMI SE, among others. Conductive foam presents an advanced alternative for EMI shielding and grounding, offering tri-axial conductivity (X, Y, Z) essential for high-speed microprocessor systems in computing, telecommunications, and aerospace. Typically composed of copper- and nickel-plated polyurethane (PU), or PU with carbon forms, it is suitable for low-cycle applications such as I/O shielding and perimeter gasketing, operating effectively within −10 to 85 °C and a 25–75% compression range [144].

**Figure 6:**
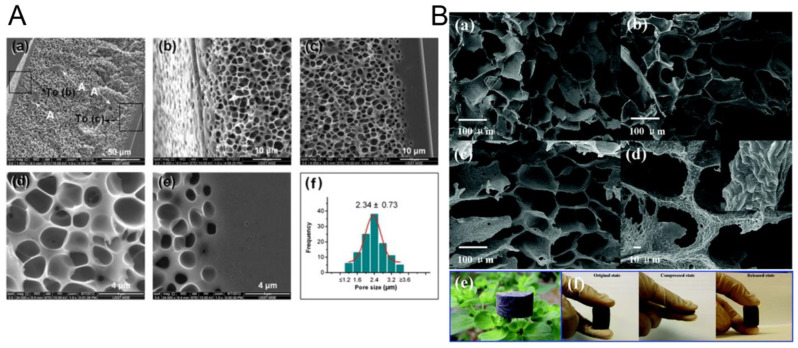
The figure (**A**), the SEM images of carbon foam (CF) different sites (**a**–**e**) and magnifications with diameter (**f**) distribution are presented [16]; (**B**) represents morphological analysis including (**a**) TPU and CPC foams incorporating graphene at (**b**) 1 wt% and (**c**) 3 wt% loadings; (**d**) a high-magnification image, (**c**) reveals the detailed cell strut architecture, with the inset illustrating uniform graphene dispersion along the strut surface; (**e**) the structural integrity of the 3 wt% graphene CPC foam is demonstrated by its ability to support a bud without deformation; and (**f**) sequential digital images of the compression cycle highlight the material’s compressibility and elastic recovery [146].

Carbon foams represent a novel class of lightweight materials with tunable thermal/electrical conductivity, high-temperature resistance, and excellent mechanical strength. Available in open- and closed-cell structures, they offer distinct properties—open-cell foams provide high permeability, while closed-cell variants feature greater strength and insulation. Due to their high EMI shielding effectiveness, carbon foams are increasingly used in defense applications to enhance stealth by attenuating radar signals [144]. Recent developments include carbon foams through coaxial electrohydrodynamic atomization (EHDA) (Figure 6A) and graphene-coated PU foams (Figure 6B) via dip-coating with cellulose-assisted dispersions, as well as PU/carbon nanostructure and polystyrene/CNT foams fabricated through innovative foaming and emulsion polymerization techniques [16,145,146].

**Figure 7:**
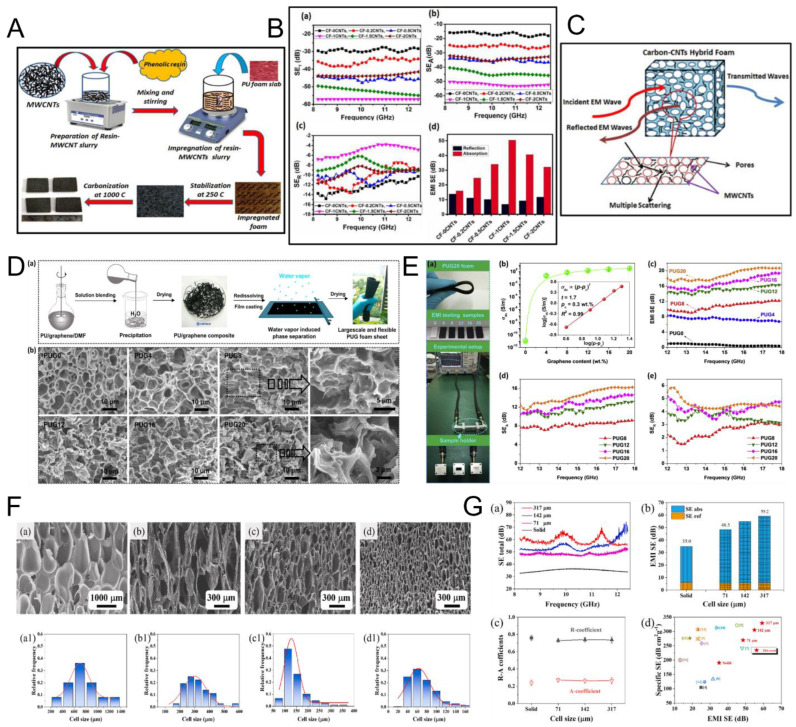
(**A**) depicts the method of preparation for the carbon-CNTs hybrid foams, while (**B**) shows the EMI SE with Total (SET) (**a**), Absorption (SEA) (**b**), Reflection (SER) (**c**) in the frequency range of 8.2–12.4 GHz, and absorption and reflection at a fixed frequency of 8.2 GHz (**d**). (**C**) is a proposed EMI SE mechanism in carbon–CNTs hybrid foam [147]. The (**D**) shows the schematic design for the preparation of PUG foams (**a**), and the SEM images of the PUG foams (**b**), while the (**E**) demonstrate the digital images of the PUG20 foam EMI with diverse contents of carbon with a used setup (**a**), as well as DC conductivity (**b**), and SE total, SE absorption (SEA), and SE reflection (SER) of PUG foams (**c**–**e**) [15]. The (**F**) represents the SEM images of PP (**a**) and PP/CNS nanocomposite foams (**b**–**d**) produced under different N_2_ content: (**a**) 1 wt%, (**b**) 0.6 wt%, (**c**) 0.8 wt%, (**d**) 1 wt%, with a size distribution (**a1**–**d1**), while the (**G**) represents EMI SE of PP/CNS in the X–band, from SE total (**a**), SE absorbance and SE reflection (**b**), R and A coefficients (**c**), and the evaluation of the EMI SE performance with previously reported CPC foams (**d**) [145].

Patle et al. produced three-dimensional fire-retardant carbon–carbon nanotube (CNT) hybrid foams for EMI shielding using a PU template [147]. Phenolic resin was blended with varying concentrations (0.2–2 wt%) of multi-walled carbon nanotubes (MWCNTs) under magnetic stirring to achieve a homogeneous slurry. PU foams were then impregnated with this mixture and subjected to carbonization at 1000 °C (Figure 7A). The resulting hybrid foams were systematically characterized in terms of morphology, structure, mechanical performance, EMI shielding efficiency, and flame retardancy. The highest EMI shielding effectiveness (SE) of −57.2 dB was recorded for the foam containing 1 wt% MWCNTs in the X-band frequency range (8.2–12.4 GHz) (Figure 7B). The shielding mechanism is presented in Figure 7C. Furthermore, the incorporation of MWCNTs significantly improved compressive strength (up to 6.5 MPa), thermal stability, and fire resistance. These multifunctional carbon–CNT hybrid foams exhibit excellent potential for EMI shielding and thermal management in aerospace and defense applications.

Li et al. developed multilayered thermoplastic polyurethane (TPU)/graphene sandwich-structured nanocomposite foams by stacking individual TPU/graphene foam layers (Figure 7D) [15]. These sandwich architectures exhibited enhanced electromagnetic wave absorption characteristics. As the foam thickness increased, reflection losses occurred due to constructive interference effects. The EMI shielding performance of the composites was evaluated in the Ku-band frequency range, revealing that shielding effectiveness improved with increasing graphene content. At a loading of 20 wt% graphene, the shielding effectiveness ranged between 17 and 21 dB, attributed to the formation of conductive networks within the matrix (Figure 7E). In contrast, the unmodified TPU was electrically insulating and allowed electromagnetic radiation to pass through. The shielding mechanisms involved absorption (SEA) and reflection (SER), significantly enhanced with an increase in graphene concentrations. Bernal et al. synthesized rigid PU nanocomposite foams incorporating multi-walled carbon nanotubes (MWCNTs), functionalized MWCNTs (f-MWCNTs), and functionalized graphene sheets (FGS) via reactive foaming, targeting applications in EMI shielding [148]. The study demonstrated that the electrical properties of the PU foams were significantly influenced by the foaming behavior, cellular morphology, and material density, which were in turn dependent on the aspect ratio, dispersion, and surface functionalization of the carbon-based nanofillers. The highest EMI shielding effectiveness (SE) was achieved with only 0.35 wt% MWCNTs, attributed to a highly interconnected conductive network, resulting in a two-order-of-magnitude increase in electrical conductivity. The corresponding EMI SE reached 27 dB, well above the 20 dB threshold typically required for commercial applications, even without normalizing by density. Wu et al. demonstrated that introducing a cellular structure into conductive polymer composites (CPCs) can significantly enhance their electrical properties [145]. They proposed an innovative method to increase cell size as a means to improve both electrical conductivity (EC) and EMI SE. Using a core-back foaming injection molding technique, they fabricated polypropylene/carbon nanostructure (PP/CNS) nanocomposite foams with a fixed void fraction, where cell size was precisely controlled by adjusting nitrogen (N_2_) content (Figure 7F). Their findings showed that increasing the cell size from 71 to 317 μm led to an increase in EC from 1.43 × 10^−3^ to 5.07 × 10^−3^ S/cm and EMI SE from 48.5 to 59.2 dB (Figure 7G). The improved conductivity was attributed to the partial alignment of carbon nanostructures and the reduced length of conductive pathways. The CPC foams also achieved a maximum specific shielding effectiveness of 329 dB·cm^2^/g, outperforming many existing CPC-based shielding materials (Table 1).

Carbon foams are a new category of lightweight materials prized for their high strength, heat resistance, and adjustable thermal/electrical conductivity. Their EMI shielding capabilities make them useful in defense applications for attenuating radar signals.

## 6. Conclusions

EMI has emerged as a significant concern owing to the growing dependence on electronic and wireless technology across almost all industries. Common sources of EMI, including communication systems, industrial machinery, and consumer electronics, negatively influence device performance and may also present threats to human health and environmental integrity. These apprehensions have propelled the necessity for more efficient and sustainable EMI shielding systems. The EMI shielding market is undergoing significant expansion, driven by the spread of 6G networks, electric vehicles, medical devices, and aerospace applications. As this industry progresses, the demand for materials that provide shielding effectiveness while reducing environmental impact also increases. Carbon-based nanomaterials—particularly graphene, carbon nanotubes, and carbon foams—emerge as more promising candidates for future EMI shielding due to their tunable electrical properties, lightweight nature, flexibility, and eco-friendly potential. Nonetheless, challenges such as large-scale synthesis, dispersion uniformity, and frequency-dependent performance must still be resolved before they can fully displace conventional metallic shields. In addressing whether carbon-based nanomaterials are indeed more promising, the answer is affirmative but with nuance. While metals still deliver better shielding effectiveness, particularly in reflection mechanisms, carbon nanomaterials offer clear advantages for next-generation applications (wearables, aerospace, and biomedical devices) where weight, flexibility, and sustainability are critical. The most impactful direction lies in carbon-based composites, which synergistically combine absorption-dominant shielding with reduced weight and environmental compatibility, aligning closely with global sustainability and technology trends.

As industries shift towards more intelligent and sustainable electronics, carbon nanomaterials are set to be crucial in mitigating EMI issues, protecting human and environmental health, and facilitating the advancement of clean, interconnected technologies.

**Figure 8:**
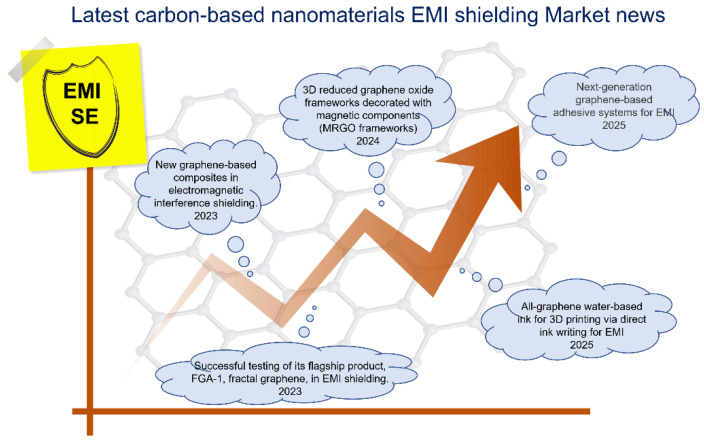
The latest news on the EMI shielding market by different EU projects/companies based on the research by Graphene-info [20].

If we look over only the past three years, the market for carbon-based nanomaterials in EMI shielding has experienced significant expansion, with various EU initiatives and organizations engaged in seeking solutions for this increasing challenge (Figure 8) [20].

## Figures and Tables

**Table 1:** Comparison of the shielding materials based on advantages, limitations and shielding efficiency.

Category	Examples	Advantages	Limitations	Shielding Effectiveness	Ref.
Metals	Cu, Ag, Ni, Al, Fe	conductivity, reflection shielding	heavy and rigid, corrosion/oxidation issues, expensive, poor flexibility and recyclability	Very high >100 dB	[123]
Metal-coated Polymers/Foams	Ni-coated PU foams, Cu-coated fabrics	lightweight, flexible, conductivity	coating adhesion issues, mechanical durability limits, complex processing, recycling difficulty	High 60–90 dB	[10]
Conductive Polymers	Polyaniline, Polypyrrole	lightweight, flexible, corrosion resistant, tunable conductivity	lower conductivity than metals, thermal instability, degradation over time	Moderate 20–60 dB	[124]
Carbon-based Nanomaterials	Graphene, CNTs, carbon foams, carbon black	lightweight, flexible, corrosion resistant electrical/thermal conductivity absorption reflection shielding environmentally sustainable, scalable in composites, coatings, inks	production cost, dispersion/agglomeration issues, shielding performance still below metals at high frequencies, long-term stability under harsh conditions	High when engineered 40–90 dB, potentially higher in composites	[125,126]

## Data Availability

Data available at Zenodo, doi:10.5281/zenodo.17244261.

## Abbreviations

**Acronyms**	**Full name**
AC	alternating current
AEDs	automated external defibrillators
AgNW	and silver nanowire
AI	Artificial Intelligence
AICDs	automatic implantable cardioverter defibrillators
AIMD	active implanted devices
BBB	blood-brain barrier
CA	Cornu Ammonis
CAGR	compound annual growth rate
CF	carbon foam
CM	common mode
CNS	central nervous system
CNT	carbon–carbon nanotube
CPCs	conductive polymer composites
CR	circadian rhythm
CVD	chemical vapor deposition
DFT	density functional theory
DM	differential mode
DTX	discontinuous transmission
EAS	electronic article surveillance
EC	electrical conductivity
ECG	electrocardiogram
EEGs	electroencephalograms
EHDA	electrohydrodynamic atomization
ELF	Extremely Low Frequency
ELF-EMFs	extremely low frequency electromagnetic fields
EM	electromagnetic
EMI	Electromagnetic interference
EMPs	electromagnetic pulses
EMW	electromagnetic wave
ESD	Electrostatic Discharge
EUT	equipment under test
FGS	functionalized graphene sheets
GO	graphene oxide
GSM	global system for mobile communications
HI	hydrogen iodide
HRV	heart rate variability
ICA	independent component analysis
ICDs	implantable cardioverter-defibrillators
IoT	Internet of Things
LISN	line impedance stabilization network
MRI	Magnetic Resonance Imaging
MW	Microwave
MWCNTs	multi-walled carbon nanotubes
PEEK	Polyether Ether Ketone
PEMF	pulsed electromagnetic field
PM	pacemaker
PP	Polyphenylene
PPA	polyethene polyamine-modified polyacrylonitrile
PPS	Polyphenylene Sulfide
PU	polyurethane
PUG	polyurethane/graphene
PWM	Pulse Width Modulation
RF	radio frequency
RF-EMF	Radiofrequency Electromagnetic Fields
RFID	radio-frequency identification
rGO	reduced graphene oxide
ROS	reactive oxidative species
SCHEER	Scientific Committee on Health, Environmental and Emerging Risks
SE	shielding effectiveness
SEM	Scanning Electron Microscopy
SET	shows the EMI SE with Total
TDMA	time division multiple access
TEM	transverse electromagnetic
TPU	thermoplastic polyurethane
VLF-EMF	very low frequency-electromagnetic field
WHO	World Health Organization
